# 10 kHz Spinal Cord Stimulation for the Treatment of Failed Back Surgery Syndrome with Predominant Leg Pain: Results from a Prospective Study in Patients from the Dutch Healthcare System

**DOI:** 10.1111/papr.12973

**Published:** 2020-12-22

**Authors:** Jan Willem Kallewaard, Ismail Gültuna, Vincent Hoffmann, Lars Elzinga, Renate Munnikes, Lisette Verbrugge, Veerle Minne, Pascalle Reiters, Jeyakumar Subbaroyan, Angela Santos, Anand Rotte, David Caraway

**Affiliations:** ^1^ Rijnstate Ziekenhuis Velp Pijncentrum Pijnbehandelcentrum Velp The Netherlands; ^2^ Albert Schweitzer Ziekenhuis Pijnbehandelcentrum Zwijndrecht The Netherlands; ^3^ Amphia Ziekenhuis Breda Pijnbehandelcentrum Breda The Netherlands; ^4^ Bravis Ziekenhuis Roosendaal Pijncentrum Roosendaal The Netherlands; ^5^ Maasstad Ziekenhuis Rotterdam Pijnkliniek Rotterdam The Netherlands; ^6^ Nevro Corp. Redwood City California U.S.A.

**Keywords:** 10 kHz SCS, failed back surgery syndrome, leg pain and VAS

## Abstract

**Introduction:**

Persistent back/and or leg pain is a common outcome after spinal surgery (otherwise known as failed back surgery syndrome [FBSS]). Studies have shown that spinal cord stimulation (SCS) at 10 kHz provides effective analgesia in FBSS patients with both back and leg pain symptoms and in those with predominant back pain. This study is the first to evaluate the therapy in FBSS patients with predominant leg pain.

**Methods:**

The safety and efficacy of 10 kHz SCS was evaluated in an uncontrolled, open‐label, prospective study of FBSS patients with predominant leg pain in the Netherlands. Follow‐ups were performed at 1, 3, 6, and 12 months post implantation.

**Results:**

Sixty out of 68 patients (88%) experienced sufficient pain relief during a stimulation trial. Of these, 58 proceeded to permanent implantation of a 10 kHz SCS system. After 12 months of treatment, 80% of patients experienced ≥ 50% reduction in baseline leg pain, and a similar proportion (76%) experienced ≥ 50% reduction in baseline back pain. At least two‐thirds of patients were also leg pain and back pain remitters (visual analog scale [VAS] ≤ 2.5 cm). The therapy was also associated with a general improvement in patients’ quality of life, as measured by secondary outcomes including disability, perception of health improvement, mental well‐being, and satisfaction. A positive impact on opioid consumption was also observed.

**Conclusions:**

Consistent with previous findings, 10 kHz SCS for the treatment of FBSS patients with predominant radicular symptoms is safe and effective and is associated with improved quality of life.

## Introduction

Low back pain is a common health complaint that has risen in prevalence during recent decades.^1^, ^2^, ^3^ During the same time frame, the rate of spinal surgery has also increased in developed countries.^4^, ^5^, ^6^, ^7^, ^8^ While many patients benefit from surgical intervention, recent studies suggest that around one in five patients experience persistent back and/or leg pain after surgery (otherwise known as failed back surgery syndrome [FBSS]).^9^, ^10^ The prevalence of FBSS among the general population is estimated to be on par with more recognized chronic pain conditions such as rheumatoid arthritis (around 0.5% to 0.6%).^11^

Traditional spinal cord stimulation (SCS) is a minimally invasive therapy that has been widely used in recent decades to treat FBSS. During traditional SCS, low‐frequency (up to 200 Hz) electrical pulses delivered to the spinal cord elicit paresthesia over the painful area to mask the sensation of pain. Two randomized controlled trials (RCTs) have confirmed the analgesic efficacy of the therapy in FBSS patients with predominant leg pain. North and colleagues randomized patients to either reoperation or traditional SCS.^12^ After 3 years, the study found that traditional SCS provided more effective pain relief than reoperation. In the PROCESS RCT, patients received either traditional SCS and conventional medical management (CMM) or CMM alone.^13^, ^14^ Over 24 months, the traditional SCS + CMM group reported improved pain relief, quality of life, and functional outcomes compared with the CMM group.

Various European bodies have assessed the evidence base for SCS in FBSS. The European Federation of Neurological Societies (EFNS) review concluded that the therapy is efficacious in this population with grade B supporting evidence.^15^ In the U.K., the National Institute for Health and Clinical Excellence (NICE) recommended the therapy as a cost‐effective treatment option for FBSS (either as an alternative to further lumbar surgery or as an adjunct to CMM).^16^ A recent systematic review by the Zorginstituut Nederland (ZIN) showed moderate evidence for neuromodulation in FBSS patients with predominant leg pain. Reimbursement in the Netherlands is strictly dependent on the ZIN guidelines.

Ten kilohertz SCS, an alternative paresthesia‐free SCS paradigm, has emerged to address the concerns with paresthesia coverage and lack of effective pain relief in significant proportion of patients. In an RCT, 10 kHz SCS demonstrated clinical benefit in patients with both back and leg pain. The SENZA‐RCT mainly comprised FBSS patients (87%) with baseline back pain and leg pain of at least 5 cm on the visual analog scale (VAS).^17^, ^18^, ^19^ The study demonstrated that 10 kHz SCS resulted in significant and sustained decrease in both back pain and leg pain up to 24 months. Moreover, pain relief was superior to traditional SCS and was associated with improved quality of life and functional capacity. Among the 10 kHz SCS group, the absence of paresthesia and stimulation‐related discomfort were additional advantages over traditional SCS. While 10 kHz SCS has demonstrated analgesic efficacy in FBSS patients with both back and leg pain and in those with predominant back pain in multiple studies in addition to the RCT,^20^, ^21^, ^22^, ^23^ the evidence is limited in FBSS patients with predominant radicular symptoms. Additional postmarket studies are required to confirm the benefits of the therapy in this population. Therefore, the present study aimed to evaluate the efficacy of 10 kHz SCS in a Dutch population of FBSS patients with predominant leg pain.

## Methods

### Study Design and Participants

This prospective, multicenter, open‐label study was conducted in five sites throughout the Netherlands. Non‐WMO (Medical Research Involving Human Subjects Act, The Netherlands) research and local ethics committee approvals were obtained at each participating site as required. The study was performed in accordance with ISO 14155‐2011; Data Protection Directive 95/46/EC; recommendations guiding physicians in biomedical research involving humans adopted by the 18th World Medical Assembly, Helsinki, Finland (1964 and later revisions); and local laws and regulations.

Patients aged 18 years or over who had provided written informed consent were screened for enrolment according to the following key eligibility criteria: diagnosed with FBSS refractory to conservative therapy and minimally invasive pain procedures for at least 3 months and indicated for SCS as per Dutch SCS guidelines, average leg pain intensity ≥ 5 cm measured on the VAS, and back pain intensity ≤ average leg pain intensity on the VAS (Table 1). Patients had also undergone a minimum of one evaluation at the investigational site prior to screening, were appropriate candidates for the required surgical procedures, had a stable pain medication regime for at least 4 weeks prior to the baseline visit, could use the study equipment, and were able to comply with study‐related procedures.

**Table 1:** Key Inclusion/Exclusion Criteria

Inclusion Criteria	Exclusion Criteria
Diagnosed with FBSS refractory to conservative therapy ≥ 3 months and indicated as per Dutch SCS guidelines	Pain in other area(s), not intended to be treated with SCS, that could confound evaluation of study endpoints
Average leg pain intensity ≥ 5 out of 10 cm on the VAS	Radiographic evidence of mechanical spinal instability requiring fusion
Average back pain intensity ≤ average leg pain intensity on the VAS	Pain that is significantly exacerbated by activity or alleviated by rest

Major exclusion criteria included pain in one or more areas, not intended to be treated with SCS, that could confound the evaluation of study endpoints; radiographic evidence of mechanical spinal instability requiring fusion; and pain significantly exacerbated by activity or alleviated by rest (Table 1). Patients were also excluded from participation if they had visceral pain in the area being treated (non‐neuropathic pain), had benefited within 30 days prior to enrolment from an interventional procedure and/or surgery to treat back and/or leg pain, had prior experience with SCS, or were not appropriate candidates for the surgical procedures or SCS therapy. Patients with evidence of an active disruptive psychological or psychiatric disorder or other known condition significant enough to impact perception of pain, compliance of intervention and/or ability to evaluate treatment outcome, as determined by psychological evaluation prior to the trial; patients with coagulation disorders; and pregnant patients were excluded from the study.

### Procedures

**Figure 1:**
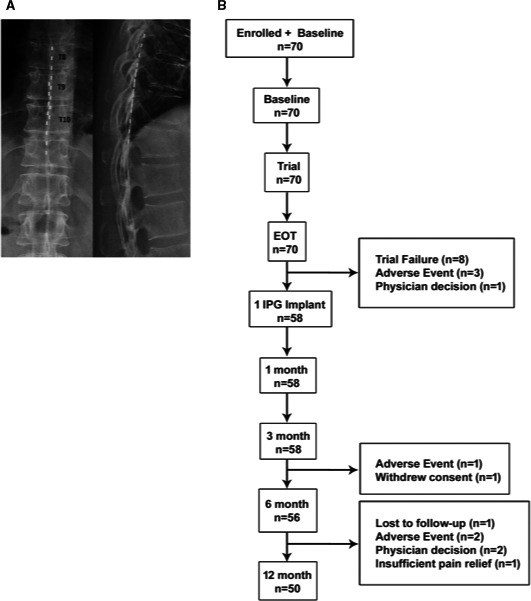
(A) Thoracic lead(s) placement. (B) Patient disposition in the study.

Eligible patients proceeded to a baseline visit followed by a temporary trial of 10 kHz SCS lasting 7 to 21 days. Each site performed the trial and implant procedure according to their usual clinical practice and following manufacturer guidelines. If no anatomical limitations were encountered, patients were implanted with two percutaneous leads staggered between the T8 and T11 vertebral levels (Figure 1A). The implanted leads were subsequently connected to a temporary external stimulation device. Impedance measurements and fluoroscopy were used to verify lead location and function. Anterior‐posterior and lateral x‐rays were also taken to confirm the final position of the leads. During the trial phase, patients received 10 kHz, 30 µs stimulation with amplitudes between 0 and 5 mA, testing outwards from the T9/T10 vertebral level until programming settings were found that provided best pain relief. Trial leads remained implanted at the end of trial period in patients who experienced ≥ 50% reduction in baseline leg pain (permanent trials). The trial stimulator was collected at the end of the trial period, and the patients either proceeded to permanent implant on the same day or were scheduled to receive a permanent implant of a 10‐kHz SCS system (Senza™ system; Nevro Corp, Redwood City, CA, U.S.A.) within 14 days. The permanent device was programmed with the optimal stimulation parameters determined during the trial phase. While detailed information on sweet spots and contacts for stimulation was not collected during the study, most patients were stimulated between mid‐T9 to mid‐T10 using the X‐rays taken at the time of the implant.

### Outcomes

Outcomes were collected at baseline and 1, 3, 6, and 12 months after permanent implantation (Figure 1B). The primary outcome measure was the responder rate at 12 months, with response defined as ≥ 50% reduction in leg pain from baseline measured on the VAS.

Secondary outcomes included responder rate prior to 12 months; the proportion of patients reporting ≥ 50% reduction in baseline back pain measured on the VAS; changes in baseline pain intensity recorded on the VAS; functional disability reported on the Oswestry Disability Index (ODI); impression of change in health evaluated using the Global Impression of Change (GIC) questionnaires for clinicians and patients; catastrophic thinking related to pain measured using the Pain Catastrophizing Scale (PCS); emotional mood status measured on the Hospital Anxiety and Depression Scale (HADS); and patient satisfaction. Medication usage and change in opioid dose following treatment were not the endpoints of the study but were collected and reported for all patients on opioids during the study.

### Statistics

Descriptive statistics were used to summarize data collected during the study. Continuous variables were summarized using the number of observations (patients), mean, standard error of the mean (SEM), minimum, median, and maximum. Categorical variables were summarized using counts and percentages. As this was a noncomparative study, the sample size was computed to reach a prespecified precision of 60% to 65% for the responder rate at 12 months, and calculated using the confidence interval formula, based on the Clopper–Pearson method. Analysis of effectiveness‐related endpoints was conducted on patients who completed each study visit. All analyses were carried out in Microsoft Excel.

## Results

### Demographics

Between April 2015 and September 2017, 70 patients were enrolled in the study (Figure 1B). The last study visit occurred in October 2018. The demographics and baseline characteristics of the enrolled patients are detailed in Table 2. Overall, patients had experienced FBSS symptoms for 2.3 years. Around half of the cohort experienced unilateral leg pain (vs. bilateral leg pain). The majority (90%) were diagnosed with intractable back and leg pain (Table 2); however, the inclusion criteria required patients to have leg pain greater than or equal to back pain at the time of enrolment, and the mean VAS score for leg pain (7.7 cm) was higher than the back pain (5.5 cm) at baseline.

**Table 2:** Demographics and Baseline Pain Characteristics

Variable	*N*	Mean ± SD	Median (Range)
Gender			
Male	27		
Female	43		
Age (years)	70	52.6 ± 11.3	51.5 (26 to 75)
Height (cm)	70	174.6 ± 9.5	173 (156 to 203)
Weight (kg)	70	81.6 ± 15.4	83 (48 to 112)
Duration of pain (years)	70	7.6 ± 7.0	4.5 (1 to 28)
Duration of FBSS (years)*	70	2.3 ± 3.8	0^†^ (0 to 18)
Location of leg pain			
Unilateral	36		
Bilateral	34		
Pain diagnosis			
Chronic intractable back and leg pain	63		
Chronic intractable leg pain	7		
Leg pain VAS (cm)		7.7 ± 2.4	
Back pain VAS (cm)		5.6 ± 1.0	

* Duration of FBSS is the approximate year of FBSS diagnosis.

^†^ A duration of 0 means a FBSS diagnosis of < 1 year.

### Trial Phase

All 70 enrolled patients underwent a temporary stimulation trial; however, two patients experienced an adverse event during the trial phase and were excluded. Of the 68 remaining patients that completed the trial, 60 (88%) experienced a reduction of at least 50% in baseline leg pain. A further two patients were excluded after the trial phase: one due to an adverse event and one due to physician decision. In total, 58 patients proceeded to receive a permanent implant.

### Longitudinal Leg and Back Pain

**Figure 2:**
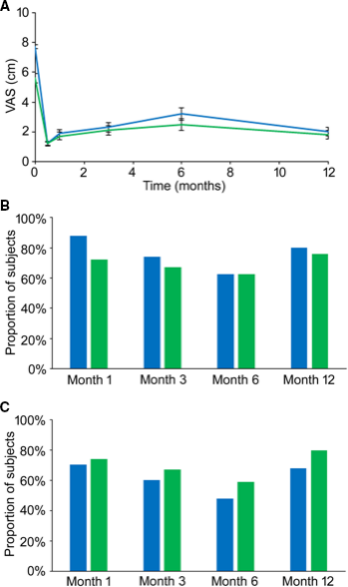
Leg and back pain relief. (A) Longitudinal leg and back pain visual analog scale (VAS) scores. (B) Leg and back responder rates for the permanent implant population (response: ≥ 50% pain relief from baseline). (C) Leg and back remitter rates for the permanent implant population (remitter: VAS ≤ 2.5 cm). Leg pain (blue) and back pain (green).

Mean leg pain VAS score (±SD) decreased from 7.7 ± 2.4 cm at baseline to 1.2 ± 2.0 cm at the end of the trial, an average reduction of 84%. A high level of leg pain relief was sustained for 12 months, with average decreases in baseline VAS score of 76%, 70%, 58%, and 75% at 1, 3, 6, and 12 months, respectively (Figure 2A).

Likewise, a high level of back pain relief was experienced during the trial phase and maintained out to 12 months (Figure 2A). Mean back pain (±SD) decreased from 5.6 ± 1.0 cm at baseline to 1.2 ± 1.6 cm at the end of the trial, an average reduction of 73%. The respective average decreases in back pain VAS score at 1, 3, 6, and 12 months were 52%, 58%, 53%, and 63%, respectively.

### Responder and Remitter Rates

At 12 months, 80% of patients (*N* = 40/50) were responders to therapy for leg pain (Figure 2B). A similar proportion (76%, *N* = 38/50) reported response for back pain (Figure 2B). In both pain regions, at least 60% of patients reported ≥ 50% pain relief throughout follow‐up.

Patients were classified as remitters if they achieved a VAS score of ≤ 2.5 cm.^17^ At 12 months, 68% (*N* = 34/50) and 80% (*N* = 40/50) of patients were leg pain and back pain remitters, respectively (Figure 2C). In both pain regions, a high proportion of patients were remitters during the follow‐up period (at least 48% for leg pain and 59% for back pain).

### Quality of Life

**Figure 3:**
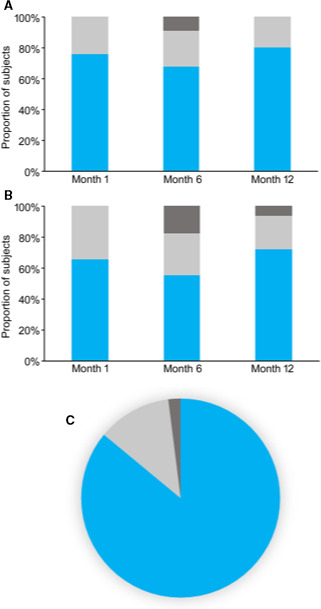
Global Impression of Change (GIC) and satisfaction. (A) Clinician‐assessed GIC (CGIC) with categorization *Very Much and Much Improved* (blue), *Minimally Improved/No Change* (light grey), and *Minimally and Much Worse* (dark grey). (B) Patient‐assessed GIC (PGIC) with categorization *Very Much and Much Improved* (blue), *Minimally Improved/No Change* (light grey), and *Minimally, Much, and Very Much Worse* (dark grey). (C) Patient satisfaction at 12 months with categorization *Very Satisfied and Satisfied* (blue), *Do not Know* (light grey), and *Dissatisfied* (dark grey).

On the GIC questionnaires collected after 12 months of treatment, 80% of patients were rated by their clinician as “very much” or “much” improved, and 72% of patients reported feeling “very much” or “much” improved (Figure 3A,B). At 12 months, 86% of the cohort indicated that they were “very satisfied” or “satisfied” with their therapy (Figure 3C).

**Figure 4:**
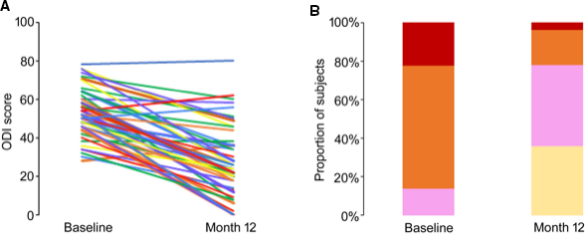
Change in disability (Oswestry Disability Index, ODI): (A) individual scores and (B) categorization *Minimal* (cream), *Moderate* (pink), *Severe* (orange), and *Crippled* (red).

Functional outcomes on the ODI are shown in Figure 4. Patients’ overall level of disability, measured using the mean ODI score, decreased from 52.4 ± 1.6 at baseline to 33.3 ± 2.5 after 1 month of treatment, an average reduction of 19.1 ± 2.0 points. After 6 and 12 months of treatment, the average decreases in ODI score were 19.7 ± 2.3 and 25.3 ± 2.3 points, respectively. Disability categorization was also evaluated according to ODI score at baseline and 12 months (minimal disability: 0 to 20; moderate disability: 21 to 40; severe disability: 41 to 60; crippled: 61 to 80; bedbound or exaggerating symptoms: 81 to 100). After 12 months of treatment, 62% of patients (*N* = 31/50) reduced their ODI score sufficiently to reclassify them from severely disabled or crippled to moderately or minimally disabled.

### Opioid Usage and Mental Status

**Figure 5:**
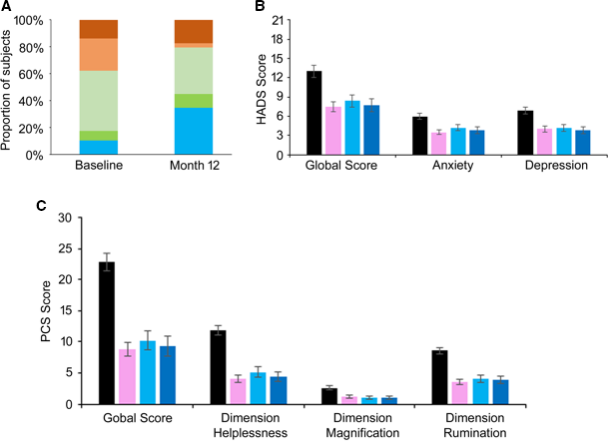
Change in opioid use and mental status. (A) Reported opiate analgesic intake from baseline until 12 months with categorization *0 MME*, *Morphine Milligram Equivalents* (blue), *PRN*, *Pro Re Nata* (dark green), *1 to 49 MME* (light green), *50 to 90 MME* (orange), and *> 90 MME* (brown). (B) Hospital Anxiety and Depression Score (HADS) at baseline (black), 1 month (pink), 6 months (light blue), and 12 months (dark blue). (C) Pain Catastrophizing Scale (PCS) at baseline (black), 1 month (pink), 6 months (light blue), and 12 months (dark blue).

Patients were classified at baseline and 12 months according to their daily intake of oral morphine equivalent (none, PRN, 1 to 49 mg/day, 50 to 90 mg/day, or > 90 mg/day; Figure 5A). Twenty‐nine out of 58 implanted patients were taking opioids at baseline or at 12 months. Of these, seven (24%) ceased intake, and a further two reduced their consumption (7%). The percentage of patients taking opioids at safe doses (none, PRN, or < 50 mg/day^24^, ^25^) increased to 80% at 12 months (Figure 5A).

Patients’ mental status, measured on the HADS scale, improved during the study compared with baseline (Figure 5B). The HADS anxiety subscale mean scores decreased from 6.0 ± 0.5 points at baseline by an average of 2.4 ± 0.3, 1.9 ± 0.4, and 2.2 ± 0.5 points after 1, 6, and 12 months of treatment, respectively. Likewise, the HADS depression subscale mean scores decreased from 6.9 ± 0.6 points at baseline by an average of 2.9 ± 0.4, 2.8 ± 0.4, and 3.2 ± 0.5 points after 1, 6, and 12 months of treatment, respectively.

A decrease in pain catastrophizing was also observed on the PCS at each follow‐up. Mean global PCS score reduced from 22.9 ± 1.4 points at baseline to 14.5 ± 1.3, 12.7 ± 1.7, and 14.8 ± 1.7 points at 1, 6, and 12 months, respectively (Figure 5C). The decrease in total PCS score reflected consistent reductions vs. baseline in all three subscales of the PCS throughout follow‐up (helplessness, magnification, and rumination).

### Safety

No unanticipated adverse device effects (UADEs) occurred during the study at any point. A total of six serious adverse events (SAEs) were reported during the 12‐month study period in five patients. All SAEs were resolved.

## Discussion

Failed back surgery syndrome is a complex and challenging pathology to treat. 10 kHz SCS is a minimally invasive intervention that provides paresthesia‐free pain relief. The therapy is used to treat a variety of chronic pain etiologies associated with trunk and/or limb pain.^26^ To date, the evidence supporting the use of the therapy in FBSS patients is predicated on those diagnosed with either both back and leg pain or predominant back pain.

To our knowledge, this is the first study to evaluate 10 kHz SCS in FBSS patients with predominant radicular symptoms (Table 3). Among our cohort, the therapy provided substantial and robust leg pain relief without compromising back pain relief. Notably, at least two‐thirds of our patients also met the remitter criteria for leg pain and back pain. The therapy was safe and well tolerated, with no UADEs reported during the study.

**Table 3:** Outcomes Reported in Prospective and Retrospective Studies Evaluating the Benefits of 10 kHz spinal cord stimulation (SCS) in failed back surgery syndrome (FBSS) Patients

Study (Reference)	Total *N*, % of FBSS Patients	FU Period	Outcomes
SENZA RCT^17^, ^18^, ^19^	90 (87%)	24 months	Baseline vs. 24 months: Average back pain relief: 68% Average leg pain relief: 66% Responder rate back pain: 77% Responder rate leg pain: 73% ODI: Minimal to moderate category increased from 9% to 65%. Severe to crippled category decreased from 91% to 35% Satisfaction^‡^: 86% Patient rated GIC^§^: 75% Clinician rated GIC^§^: 78% Reduced/eliminated opioids: 36%*
Prospective, two‐center study in Europe^21^	72 (79%)	24 months	Baseline vs. 24 months: Average back pain relief: 61% Average leg pain relief: 57% Responder rate back pain: 60% Responder rate leg pain: 71% Average reduction in ODI score: 27% Satisfaction^‡^: 81% Average reduction in sleep disturbances at night: 62% Reduced/Eliminated opioids: 72%
Single‐center, prospective study^22^	21 (100%)	12 months	Baseline vs. 12 months: Average pain relief: 54% Responder rate pain: 67% Reduced/eliminated opioids: 65%
Retrospective, multicenter, review^27^	1660 (NR)	12 months	At last follow‐up: Average pain relief: 63% Responder rate: 74% Decreased medication: 32% Improvement in function: 72% Improvement in sleep: 68%
Single center retrospective study^30^	32 (NR)	12 months	Baseline vs. 12 months: Average back pain relief: 46% Average leg pain relief: 51% Responder rate: not reported Reduced/eliminated opioids: 71% Reduction in interventional procedures: 72%
Single‐center retrospective case series^23^	99 (94%)	12 months	Baseline vs. 12 months^†^: Average back pain relief: 53% Average leg pain relief: 53% Responder rate back pain: 56% Responder rate leg pain: 59% Patient rated GIC^§^: 83%

FBSS, failed back surgery syndrome; FU, follow‐up; Global Impression of Change, GIC, NR, not reported; ODI, Oswestry Disability Index.

* 12‐month follow‐up data.

^†^ *N* = 69 for patients with > 12 months follow‐up.

^‡^ Satisfied or very satisfied.

^§^ Moderately better, better, or a great deal better.

Our 12‐month pain relief outcomes are consistent with previous studies of the therapy that included FBSS patients with other pain patterns. The SENZA‐RCT enrolled patients with both leg pain and back pain ≥ 5 cm on the VAS.^17^, ^18^ In the 10 kHz SCS group, most patients (87%) had undergone previous spinal surgery, and average baseline leg pain and back pain VAS scores were 7.1 and 7.4 cm, respectively. The study reported responder rates in the 10 kHz SCS group of 79% in both pain areas at 12 months, along with an average decrease in mean baseline leg pain and back pain of 5.0 and 4.9 cm, respectively. Two‐thirds of the group also achieved remitter status in leg pain and back pain (VAS ≤ 2.5 cm).

In the prospective, single‐arm SENZA‐EU study of 10 kHz SCS in predominant back pain, the majority of implanted patients had a history of spinal surgery (79%).^20^, ^21^ Patients reported average baseline leg pain and back pain scores of 5.4 and 8.4 cm, respectively. At 12 months, 65% and 70% of the cohort were leg pain and back pain responders, respectively, while mean baseline leg pain and back pain decreased by an average of 3.4 cm and 5.6 cm, respectively. A smaller, single‐arm, prospective study of the therapy in FBSS patients with predominant back pain also reported a high responder rate after 12 months of treatment (67%), along with a decrease in average VAS score of 4.7 cm from a baseline mean of 8.7 cm.^22^

Stauss and colleagues also evaluated 10 kHz SCS in a large, retrospective, real‐world study of chronic back and leg pain patients.^27^ Surgical history was not reported; however, as FBSS is the most common indication for SCS implantation,^28^, ^29^ the cohort is likely to have included a high proportion of FBSS patients. The authors reported a combined responder rate of 78% for back and leg pain at 12 months, as well as a reduction in median pain intensity among the responder group of 5.0 points from a baseline median of 8.0 points (numerical rating scale).

Among chronic pain patients, pain relief is a critical measure of therapy effectiveness. However, the impact of a therapy on patients’ lives beyond pain relief is also an important consideration. After 12 months of 10 kHz SCS, patients in the current study reduced their level of disability and reported a perceived improvement in health. Furthermore, a third of the patients that took opioids at some point during the study reduced or eliminated their intake. Previous studies of 10 kHz SCS in FBSS patients have documented similar benefits.^19^, ^21^, ^22^, ^27^, ^30^

In the SENZA‐RCT, 70% of the 10 kHz SCS group reduced their ODI classification at 12 months, while quality of life in the cohort increased across various scales.^19^ In addition, 35% of patients reduced or eliminated their opioid intake. The SENZA‐EU study found a functional improvement after 24 months, as well as reduced opioid consumption.^21^ A smaller, single‐arm, prospective study of the therapy in FBSS patients with predominant back pain also reported functional improvement and reduced opioid intake after 12 months.^22^ In the large, retrospective study by Stauss et al.,^27^ a high proportion of the cohort indicated improved function (72%) and quality of life (90%), and a third of the cohort reduced their medication intake.

Some of the wider benefits of 10 kHz SCS in our cohort are further reflected in their improved mental well‐being. Average anxiety and depression scores both decreased on the HADS scale, and pain catastrophizing reduced on the PCS. These outcomes may be important because affective aspects such as depression, anxiety, and catastrophizing are often associated with elevated levels of pain and disability in chronic pain patients.^31^, ^32^, ^33^, ^34^, ^35^, ^36^

Although not evaluated in our study, another important benefit of 10 kHz SCS is the absence of uncomfortable stimulation arising from postural changes, enabling patients to carry out routine daily activities with a high level of comfort. Around 95% of 10 kHz SCS patients in the SENZA‐RCT reported driving and sleeping with stimulation switched on.^19^ Stauss et al.^27^ corroborated this finding in their large retrospective study. In contrast, less than two‐thirds of traditional SCS patients in the SENZA‐RCT indicated being able to do the same,^18^, ^19^ and almost half reported uncomfortable stimulation.^17^ Being able to drive and sleep with minimal pain and discomfort are meaningful everyday aspects for patients. Driving is especially crucial for those who wish to return to work after treatment.

Overall, the present study showed promising outcomes in this group of Dutch FBSS patients with predominant radicular symptoms. To our knowledge, this study is the first to evaluate 10 kHz SCS in this patient group. However, our study lacked a control group and randomization, which could lead to selection bias in the patient population. Moreover, the study enrolled patients with predominant leg pain, and the patients also had back pain albeit with comparatively less intensity. The study could not isolate the patients who had only leg pain because the majority of FBSS patients have back pain along with leg pain with varying intensity. However, in all subjects, leg pain was the predominant complaint following back surgery. Consequently, our results should be interpreted within this context.

## Conclusions

Failed back surgery syndrome is a complex pathology that leaves patients with chronic back and/or radicular pain that is often refractory to conventional treatment. The present study prospectively evaluated 10 kHz SCS in FBSS patients with predominant leg pain and found the treatment to be safe and effective. The findings demonstrate that 10 kHz SCS may be a useful therapeutic option to treat the diverse range of back and leg pain symptoms experienced by FBSS patients.

## Conflict of Interests

J.K.W. is a consultant for Nevro Corp. A.R., A.S., D.C., J.S., P.R., and V.M. are employees of Nevro Corp. Rest of the authors have no conflicts to disclose.
